# Comparative analysis of risk factors for retinopathy of prematurity in single and multiple birth neonates

**DOI:** 10.1186/s40942-024-00536-6

**Published:** 2024-02-27

**Authors:** Mohammadkarim Johari, Afshin Karimi, Mohammadreza Mojarad, Mojtaba Heydari

**Affiliations:** Poostchi Ophthalmology Research Center, Department of Ophthalmology, School of Medicine, Shiraz University of Medical Sciences, Shiraz, Iran

**Keywords:** Retinopathy of prematurity, ROP, Multiple birth, Multiple gestation, Twin, Triplet, Risk factor

## Abstract

**Aim:**

To conduct a comparative analysis of risk factors for retinopathy of prematurity (ROP) in single- and multiple-born neonates.

**Methods:**

In a retrospective evaluation of 521 premature neonates, encompassing singletons, twins, and triplets born at or before 34 weeks of gestational age with a birthweight of less than 2000 g and who completed the ROP screening program, between 2020 and 2023, in outpatient referral ROP screening clinic affiliated by Shiraz University of Medical Sciences, were included. Neonates with the eligibility criteria were enrolled in the screening program from 28 days old age and followed up to discharge or treatment based on national ROP screening guideline. Data on ROP severity, outcome, treatment modality, and risk factors, including gestational age (GA), birth weight (BW), sex, duration of neonatal intensive care unit (NICU) admission, oxygen supplementation, mechanical ventilation, blood transfusion, method of delivery, and maternal and neonatal comorbidities, were extracted and compared between premature neonates from singleton and multiple births.

**Results:**

The analysis of the ROP severity distribution revealed 238 neonates (45.7%) with low-risk (type 2 prethreshold ROP or less severe) ROP and 16 (3.1%) with high-risk (type I prethreshold ROP or more severe) ROP who underwent treatment. According to the comparative analysis of risk factors in neonates with ROP requiring treatment, multiple birth neonates exhibited significantly greater GA (27.50 ± 3.27 vs. 30.00 ± 2.00 vs. 31.14 ± 0.38 weeks, *p* = 0.032 for singletons, twins and triplets, respectively); greater BW (861.67 ± 274.62 vs. 1233.33 ± 347.75 vs. 1537.14 ± 208.86 g, *p* = 0.002); and shorter duration of NICU admission (60.17 ± 21.36 vs. 34.00 ± 12.17 vs. 12.00 ± 6.32 days, *p* = 0.001) and oxygen supplementation (47.33 ± 16.57 vs. 36.00 ± 8.49 vs. 4.60 ± 2.41 days, *p* = 0.001). There was no significant difference between single-born neonates and multiple-born neonates regarding the prevalence of other risk factors. Multiple-born neonates with no ROP and low risk ROP showed significantly lower GA and BW compared to singletons (*p* < 0.001).

**Conclusion:**

Multiple gestation neonates may develop high-risk ROP requiring treatment at a greater gestational age and birth weight and at a lower duration of oxygen supplementation and NICU admission compared to the single birth neonates. This pattern prompts a reevaluation of screening criteria, suggesting a potential need to consider multiple birth neonates with lower traditional risk factors in screening programs. This pattern should be further evaluated in larger populations of multiple born premature neonates.

## Introduction

Retinopathy of prematurity (ROP) represents a formidable challenge in the realm of neonatal medicine, the prevalence of which has increased in recent years, posing a substantial burden on healthcare systems globally [1]. This potentially blinding ocular disorder primarily afflicts premature infants, particularly those with low birth weight and underdeveloped retinas [2]. As a leading cause of visual impairment in children [2, 3], the intricate etiology and multifaceted risk factors for ROP necessitate comprehensive exploration to improve prevention and treatment strategies [4].

Understanding the intricate web of risk factors associated with ROP is paramount in the development of effective screening programs [5–7]. The multifaceted nature of ROP, influenced by variables such as gestational age, birth weight, maternal and neonatal comorbidities, and medical interventions such as oxygen supplementation and blood transfusion, underscores the importance of discerning specific risk profiles [4, 8, 9]. These risk factors serve as critical indicators, guiding the formulation of targeted screening strategies tailored to the unique needs of neonates [10].

Despite concerted efforts to discern and mitigate the risk factors associated with ROP, there is a noteworthy lacuna in our understanding of the potential variations in the manifestations of ROP among multiple births [11–13]. The intricate interplay of factors such as gestational age, birth weight, and medical care nuances unique to multiple births may engender distinct risk profiles for ROP [14]. However, the current body of literature predominantly focuses on general risk factors, with only a limited number of studies systematically comparing the intricacies between single- and multiple-born neonates. This research aimed to bridge this crucial gap by performing a meticulous analysis of various risk factors contributing to the development of ROP in neonates, with a specific emphasis on discerning disparities between single and multiple births.

## Methods

### Study Design

This retrospective study aimed to compare the risk factors for ROP in singleton-born neonates and multiple-born neonates born in southern Iran between 2020 and 2023. This study utilized both observational and analytical approaches to comprehensively investigate the associations between various risk factors and the severity of ROP and to compare the findings between singleton-born neonates and multiple-born neonates.

### Study population

**Fig. 1:**
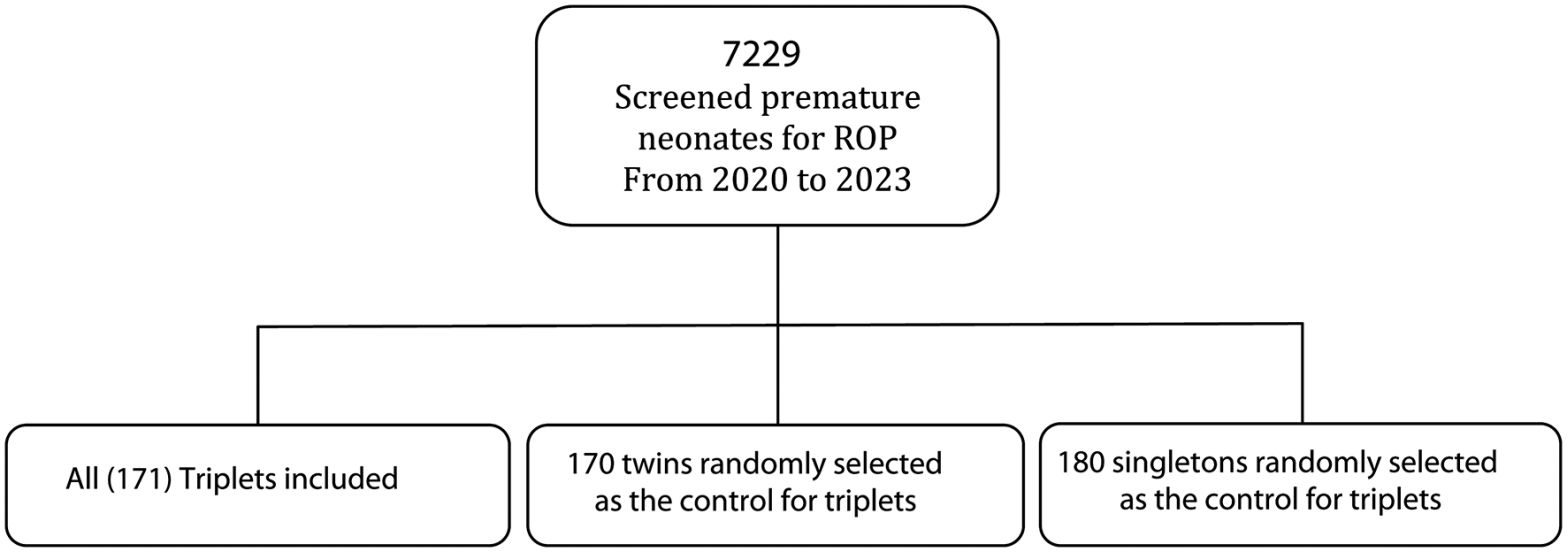
The included singletons, twins and triplets premature neonates in the analysis

The study included single, twin and triplet neonates born at or before 34 weeks of gestational (GA) age and those with a birth weight (BW) of less than 2000 g in southern Iran; these patients were referred for ROP screening at the Poostchi ROP Clinic affiliated with Shiraz University of Medical Sciences. Neonates who did not survive until ROP screening or were not included in mandatory ROP screening were excluded from the study. Neonates with the eligibility criteria were enrolled in the screening program from 28 days old age and followed up to discharge or treatment based on national ROP screening guideline. From a pool of 7,229 screened neonates, all eligible triplets meeting the inclusion criteria were enrolled. An approximately equal number of twins and singletons were randomly selected to serve as the control groups for comparison with the triplets, facilitating the analysis of risk factors across the triplet, twin, and singleton populations (Fig. 1). Regarding the severity of ROP, we classified our neonates as not having ROP (stage 0, zone 3 ROP), low risk (type 2 prethreshold ROP or less severe) or high risk (ROP, which needed treatment, type I prethreshold ROP, threshold ROP, or aggressive posterior ROP) [15].

### Data collection

The data were collected from 521 premature neonates (180 singletons, 170 twins and 171 triplets) who completed ROP screening. The collected variables included GA, corrected GA, birth weight, sex, oxygen supplementation, duration of neonatal intensive care unit (NICU) admission, mechanical ventilation, blood transfusion, method of delivery, cause of preterm labor, and specific ROP-related parameters such as ROP stage, zone, plus disease, and laser or intravitreal bevacizumab (IVB) treatment.

### Data analysis

Descriptive statistics were employed to summarize the characteristics of the neonates, presenting means, percentages, and distributions of the collected variables. Inferential statistics, including chi-square tests and t tests, were used to assess associations and differences between categorical and continuous variables among single- and multiple-born neonates.

### Ethical considerations

The study protocol was approved by the Shiraz University Medical Sciences Ethics Committee (approval number: IR.SUMS.REC.1402.214), and informed consent was obtained from all the parents or legal guardians of the neonates included in the study.

## Results

### ROP severity distribution

The analysis of the ROP severity distribution among single- and multiple-born neonates revealed noteworthy patterns (Table 1). Among the 521 neonates studied, a total of 267 (51.2%) had no signs of ROP, with slightly greater percentages of singleton births (56.1%) than twins (49.4%) or triplets (48.0%). In the category of low-risk ROP (type 2 ROP or less severe), 238 neonates (45.7%) exhibited signs of low-risk ROP, with a relatively balanced distribution across the birth scenarios. Predictably, high-risk ROP requiring treatment (type I ROP or more severe) was less prevalent, with an overall incidence of 3.1%. Notably, the occurrence of severe ROP was more prominent in twins (48.0%) than in both single and triplet births.

**Table 1:** Retinopathy of prematurity severity distribution in single- and multiple-born neonates

Severity	Singles N(%)	Twins N(%)	Triplets N(%)	Total N(%)
No ROP	101 (56.1)	84 (49.4)	82 (48.0)	267 (51.2)
Low risk ROP (type 2 ROP or less severe)	73 (40.6)	83 (48.8)	82 (48.0)	238 (45.7)
High risk ROP (Treated) (type I ROP or more severe)	6 (3.3)	3 (1.7)	7 (4.1)	16 (3.1)
Total	180 (100%)	170 (100%)	171 (100%)	521 (100%)

### ROP risk factor distribution

Table 2 presents a comprehensive comparative analysis of ROP risk factors among singletons, twins, and triplets. Gestational age varied across the groups, with singles having a mean gestational age of 33.72 weeks, twins at 33.15 weeks, and triplets at 32.47 weeks. Similarly, birth weight followed a decreasing trend, with singles having the highest mean weight at 2114.63 g, followed by twins (1849.74 g) and triplets (1641.78 g). Noteworthy differences were observed in the duration of NICU admission and oxygen support, where single patients had a longer NICU stay (13.63 days) and longer duration of oxygen support (7.36 days). The incidence of sepsis was slightly greater for singleton pregnancies (5.6%) than for twin pregnancies (0.6%) or triplet pregnancies (2.3%). The female to male ratio was 230/291 (44.1-55.9%) across all groups, with a greater percentage of females being twins and triplets (Table 1). Blood transfusion and maternal comorbidity rates were relatively consistent across the groups, while neonatal comorbidity rates were comparable among the three birth scenarios.

**Table 2:** The distribution of retinopathy of prematurity risk factors in single- and multiple-born neonates

	Singles N(%) Mean ± SD (95%CI)	Twins N(%) Mean ± SD (95%CI)	Triplets N(%) Mean ± SD (95%CI)	Total N(%) Mean ± SD (95%CI)
Gestational age (weeks)	33.72 ± 2.83 (33.31–34.14)	33.15 ± 1.80 (32.88–33.43)	32.47 ± 1.63 (32.23–32.72)	33.13 ± 2.22 (32.94–33.32)
Birth weight (gram)	2114.63 ± 683.90 (2014.05-2215.22)	1849.74 ± 369.45 (1793.80-1905.67)	1641.78 ± 319.53 (1593.55-1690.02)	1873.00 ± 526.03 (1827.731918.28)
Duration of NICU admission (days)	13.63 ± 14.87 (11.44–15.81)	10.91 ± 9.71 (9.44–12.38)	14.14 ± 10.44 (12.56–15.72)	12.91 ± 12.01 (11.88–13.94)
Duration of oxygen support (days)	7.36 ± 10.72 (5.58–9.15)	5.76 ± 7.61 (4.38–7.13)	6.03 ± 5.86 (4.86–7.20)	6.46 ± 8.60 (5.57–7.35)
Sepsis	10 (5.6%)	1 (0.6%)	4 (2.3%)	15 (2.9%)
Gender (Female/Male)	73/107 (40.6%/59.4%)	73/97 (42.9%/57.1%)	84/87 (49.1%/50.9%)	230/291 (44.1-55.9%)
Blood transfusion	28 (15.6%)	19 (11.2%)	26 (15.2%)	73 (14%)
Maternal comorbidity	39 (21.7%)	25 (14.7%)	30 (18.9%)	94 (18.0%)
Neonatal comorbidity	19 (10.6%)	14 (8.2%)	18 (10.5%)	51 (9.8%)

### Comparative analysis of risk factors in neonates with different ROP severity

**Fig. 2:**
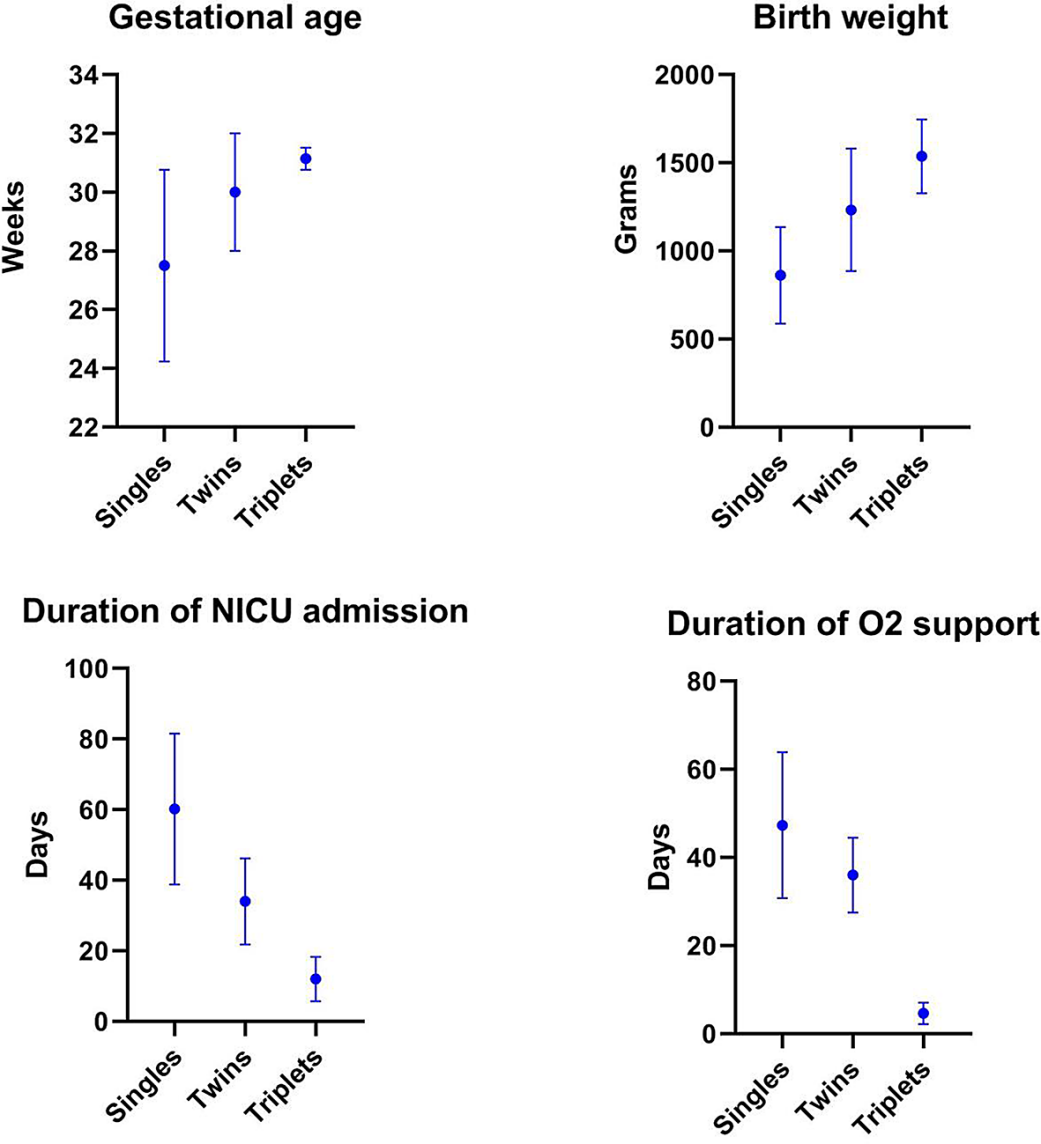
Comparative analysis of risk factors in neonates with severe ROP. The graphs show a consistent pattern of higher gestational age and birth weight as well as shorter durations of NICU admission and oxygen support in patients needing treatment for severe ROP (type I ROP or more severe) among multiple birth neonates compared to singletons

The comparative analysis of risk factors in neonates with high-risk retinopathy of prematurity (ROP), those necessitating treatment for type I ROP or more severe conditions, underscores noteworthy disparities among singletons, twins, and triplets (Fig. 2). Notably, multiple birth neonates exhibited significantly greater gestational age (GA) levels, with twins at 30.00 weeks and triplets at 31.14 weeks, than with a singleton mean of 27.50 weeks (*p* = 0.032). Birth weight variations were also evident, with multiple birth neonates having notably greater mean weights (*p* = 0.002): single at 861.67 g, twin at 1233.33 g, and triplet at 1537.14 g. Moreover, the analysis revealed a consistent pattern of a shorter duration of NICU admission (*p* = 0.001) and a shorter duration of oxygen support (*p* = 0.001) in multiple birth neonates. Multiple-born neonates with no ROP and low risk ROP showed significantly lower GA and BW compared to singletons (*p* < 0.001) (Table 3).

**Table 3:** Comparing risk factors for retinopathy of prematurity (ROP) in singleton and multiple birth neonates with different severity of ROP

Risk factors	Multiple birth	No ROP Mean ± SD	p	Low risk ROP Mean ± SD	p	High Risk ROP Mean ± SD	p
Gestational age (weeks)	Singles	34.73 ± 2.28	< 0.001	32.84 ± 2.54	0.027	27.50 ± 3.27	0.032
	Twins	33.42 ± 1.31		33.00 ± 2.10		30.00 ± 2.00	
	Triplets	32.90 ± 1.58		32.16 ± 1.61		31.14 ± 0.38	
Birth weight (grams)	Singles	2324.79 ± 667.54	< 0.001	1926.85 ± 556.88	< 0.001	861.67 ± 274.62	0.002
	Twins	1939.52 ± 349.37		1781.14 ± 357.62		1233.33 ± 347.75	
	Triplets	1694.27 ± 337.28		1598.23 ± 302.72		1537.14 ± 208.86	
Duration of NICU admission (days)	Singles	9.36 ± 8.77	0.005	15.71 ± 14.16	0.191	60.17 ± 21.36	< 0.001
	Twins	8.06 ± 7.60		12.96 ± 10.00		34.00 ± 12.17	
	Triplets	12.57 ± 10.86		15.89 ± 10.10		12.00 ± 6.32	
Duration of oxygen support (days)	Singles	5.01 ± 4.98	0.380	6.44 ± 7.06	0.441	47.33 ± 16.57	0.001
	Twins	4.03 ± 5.34		6.54 ± 7.39		36.00 ± 8.49	
	Triplets	3.98 ± 3.93		8.06 ± 6.87		4.60 ± 2.41	

## Discussion

In the present study encompassing 521 premature neonates, we discovered significant variations in risk factors associated with high-risk ROP among singletons, twins, and triplets, particularly those necessitating treatment for type I ROP or more severe conditions. Notably, multiple birth neonates exhibited greater gestational age and birth weights than singletons did, as did neonates with consistently shorter durations of NICU admission and oxygen support.

The observed heightened risk of ROP requiring treatment among multiple birth neonates despite higher GA and birth weight and shorter duration of admission and oxygen supplementation raises intriguing questions about the interplay of various factors influencing ROP development. One plausible explanation could be the inherent biological variability among multiples, leading to divergent developmental trajectories [16, 17]. Despite similar GAs and birth weights, individual multiples may experience distinct physiological challenges in the neonatal period, possibly related to differences in placental function, the intrauterine environment, or genetic factors [18]. These subtle variations could contribute to variations in the maturation and vulnerability of the developing retina, potentially explaining the unexpected prevalence of severe ROP in this subgroup.

To contextualize the study findings, they should be interpreted against the previous studies that explored the relationship between multiple gestations and ROP. In a study by Riazi-Esfahani et al., no significant difference was observed between multiple-born neonates and matched singletons in terms of the frequency and severity of ROP [19]. This finding aligns with our results that the prevalence of ROP was not significantly different among singletons and multiple birth neonates. Additionally, Dos Santos Motta et al. reported a greater frequency of ROP at any stage in twins and triplets but not for threshold disease, suggesting a potential link between multiple pregnancies and ROP [20]. However, our findings provide a more comprehensive evaluation of multiple risk factors, revealing specific differences in gestational age, birth weight, and neonatal care duration among various birth scenarios.

Another study by Yau et al. in a Chinese population revealed that younger gestational age, lighter birth weight, and various neonatal complications were independent risk factors for ROP in multiple-gestation infants [21]. While our study aligns with these risk factors, it further contributes by scrutinizing a broader spectrum of variables and directly comparing these factors among singletons, twins, and triplets. A study by Petriçli et al. focused on extremely premature infants and reported no statistically significant increase in the risk of any stage of ROP or type I ROP for multiple-born infants compared to single-born infants [22]. However, our study expands upon these findings by examining specific risk factors, such as gestational age, birth weight, and NICU admission duration, providing a more nuanced understanding of the disparities among birth scenarios. While these prior studies contributed valuable insights, our research stands out by comprehensively analyzing a diverse set of risk factors and directly comparing their prevalence and impact among singletons, twins, and triplets, thereby advancing our understanding of ROP in the context of multiple births.

Nonetheless, our study has certain limitations. The relatively low number of patients with high-risk ROP (3.1% among the studied neonates) poses a challenge in drawing definitive conclusions about this specific subgroup. The limited prevalence of high-risk ROP may restrict the statistical power and precision of our analyses, potentially hindering the generalizability of findings related to severe ROP requiring treatment. Additionally, the retrospective design employed in this study inherently hinders the establishment of causal relationships between identified risk factors and ROP development, as temporal sequences cannot be reliably discerned. Furthermore, the study’s focus on neonates referred for ROP screening at a single clinic may introduce selection bias, potentially limiting the generalizability of our findings to a broader population. Future research with larger cohorts and longitudinal designs and involving more diverse clinical settings may offer a more comprehensive understanding of these complex relationships.

The findings of our study hold significant clinical implications, particularly in the context of revising screening thresholds for ROP, especially for multiple births. The observed elevated risk of severe ROP in multiple birth neonates despite having a higher GA and BW challenges the existing screening criteria. Given that multiple birth neonates in our study exhibited increased vulnerability to severe ROP even with seemingly favorable GA and BW, reconsideration of screening thresholds may be warranted. A potential revision could involve adjusting the criteria for initiating ROP screening to accommodate the unique risk profile of multiple patients, ensuring that their increased susceptibility to severe ROP is adequately addressed. Such revisions might entail raising the threshold levels for GA and BW in multiple births, reflecting the distinct developmental trajectories of these neonates and enhancing the early detection and management of ROP in this high-risk population.

In conclusion, our study, encompassing 521 singletons, twins, and triplets, revealed a noteworthy trend in the development of high-risk ROP requiring treatment among multiple birth neonates. Multiple gestation neonates exhibiting higher gestational age and birth weight, coupled with shorter durations of admission and oxygen support, may still manifest an increased susceptibility to severe ROP compared to their singleton birth counterparts. This pattern prompts a reevaluation of screening criteria, suggesting a potential need to consider multiple birth neonates with lower traditional risk factors in screening programs. Recognizing the unique vulnerabilities of this population could pave the way for more targeted and effective preventive measures, ensuring that neonates born as multiples receive timely interventions to mitigate the risk of high-severity ROP.

## Data Availability

All data are available on rational request from corresponding author.
